# The M1 form of tumor-associated macrophages in non-small cell lung cancer is positively associated with survival time

**DOI:** 10.1186/1471-2407-10-112

**Published:** 2010-03-25

**Authors:** Junliang Ma, Lunxu Liu, Guowei Che, Nanbin Yu, Fuqiang Dai, Zongbing You

**Affiliations:** 1 Department of Thoracic and Cardiovascular Surgery, West China Hospital, Sichuan University, Chengdu 610041, China; 2 The Third People's Hospital of Zigong City, Sichuan Province, China; 3 Daping Hospital, the Third Military Medical University, Chongqing City, China; 4 Departments of Structural & Cellular Biology and Orthopaedic Surgery, Tulane Cancer Center, LCRC, Tulane Center for Aging, Tulane University School of Medicine, New Orleans, LA 70112, USA

## Abstract

**Background:**

Tumor-associated macrophages (TAMs) play an important role in growth, progression and metastasis of tumors. In non-small cell lung cancer (NSCLC), TAMs' anti-tumor or pro-tumor role is not determined. Macrophages are polarized into M1 (with anti-tumor function) and M2 (with pro-tumor function) forms. This study was conducted to determine whether the M1 and M2 macrophage densities in NSCLC are associated with patient's survival time.

**Methods:**

Fifty patients with an average of 1-year survival (short survival group) and 50 patients with an average of 5-year survival (long survival group) were included in this retrospective study. Paraffin-embedded NSCLC specimens and their clinicopathological data including up to 8-year follow-up information were used. Immunohistochemical double-staining of CD68/HLA-DR (markers for M1 macrophages) and CD68/CD163 (markers for M2 macrophages) was performed and evaluated in a blinded fashion. The M1 and M2 macrophage densities in the tumor islets, stroma, or islets and stroma were determined using computer-aided microscopy. Correlation of the macrophage densities and patient's survival time was analyzed using the Statistical Package for the Social Sciences.

**Results:**

Approximately 70% of TAMs were M2 macrophages and the remaining 30% were M1 macrophages in NSCLC. The M2 macrophage densities (approximately 78 to 113 per mm^2^) in the tumor islets, stroma, or islets and stroma were not significantly different between the long survival and short survival groups. The M1 macrophage densities in the tumor islets (approximately 70/mm^2^) and stroma (approximately 34/mm^2^) of the long survival group were significantly higher than the M1 macrophage densities in the tumor islets (approximately 7/mm^2^) and stroma (13/mm^2^) of the short survival group (P < 0.001 and P < 0.05, respectively). The M2 macrophage densities were not associated with patient's survival time. The M1 macrophage densities in the tumor islets, stroma, or islets and stroma were positively associated with patient's survival time in a univariate analysis (P < 0.01 or 0.001). In a multivariate Cox proportional hazards analysis, the M1 macrophage density in the tumor islets was an independent predictor of patient's survival time.

**Conclusions:**

The M1 macrophage density in the tumor islets is an independent predictor of survival time in NSCLC patients.

## Background

Non-small cell lung cancer (NSCLC) remains the most common cause of cancer-related death worldwide. Metastasis may have occurred at the time of initial diagnosis, even at a very early stage such as stage IA. That explains why the five-year survival rate is approximately 67% for patients with stage IA NSCLC after putatively curative surgical resection [1]. Tumor cells use multiple mechanisms to invade extracellular matrix and metastasize to distant organs. The interaction between the tumor cells and stromal cells in the tumor microenvironment plays an important role in tumor growth and metastasis. Macrophages are prominent stromal cells in this interaction. They secret a variety of growth factors, cytokines, chemokines, and enzymes that regulate tumor growth, angiogenesis, invasion, and metastasis [2].

Recently, it has been well recognized that tumor-associated macrophages (TAMs) are not homogenous [3]. Microlocalization, in terms of where macrophages are observed under a microscope, is an important factor for prognosis. Increased number of macrophages within the tumor islets confers a marked survival advantage, whereas increased number of macrophages in the tumor stroma is associated with poor prognosis in NSCLC [4]. In addition, macrophages are polarized into two functionally distinct forms M1 and M2, mirroring the Th1 and Th2 nomenclature of T cells [3]. Differentiation of the M1 macrophages is induced by interferon-γ, lipopolysaccharides, tumor necrosis factor (TNF) α, and granulocyte-monocyte colony-stimulating factor. The M1 macrophages produce high levels of interleukin (IL)-12, IL-23, TNFα, IL-1, IL-6, CXC ligand 10 (CXCL10), inducible nitric oxide synthase (iNOS), human leukocyte antigen (HLA)-DR, and reactive oxygen and nitrogen intermediates [3,5-7]. Differentiation of the M2 macrophages is induced by IL-4, IL-10, IL-13, IL-21, activin A, immune complexes, and glucocorticoid [3]. The M2 macrophages express high levels of IL-10, IL-1 receptor antagonist, CC ligand 22 (CCL22), scavenger, mannose receptor, galactose receptor, arginase I, and CD163 antigen [3,8]. Ohri et al recently reported that the M1 macrophage density in the tumor islets is positively associated with extended survival of NSCLC patients [9].

We have previously reported that the number of TAMs in the tumor islets and the ratio of TAMs in the tumor islets versus stroma are positively associated with survival time in patients with NSCLC [10]. In this study, we further determined that the M1 form of TAMs is an independent prognostic factor in patients with NSCLC.

## Methods

### Study population

This study was approved by the Institutional Review Board of West China Hospital, Sichuan University. The procedures to obtain human lung cancer tissues and follow-up information are in accordance with the Ethical Principles for Medical Research Involving Human Subjects as formulated in the World Medical Association Declaration of Helsinki (revised in 2008). All specimens were obtained from the archives of formalin-fixed, paraffin-embedded tissue blocks in the Department of Thoracic and Cardiovascular Surgery, West China Hospital, Sichuan University. The lung cancer tissues were collected from surgeries performed from June, 1999 to August, 2001. The patients were followed up until December, 2007, through outpatient visits and/or correspondences to family members. Fifty patients with long survival time (1972.0 ± 78.0 days) and 50 patients with short survival time (351.8 ± 32.5 days) were included in this retrospective study. The inclusion criteria were: (1) follow-up data were complete; and (2) paraffin blocks were available; and (3) without pre-operative chemotherapy or radiotherapy. All of the cases that satisfied the inclusion criteria were included in this study. Histological evaluation was based on the World Health Organization criteria. Tumor stage was evaluated according to the International Union against Cancer TNM classification system. The clinicopathological characteristics were summarized in Table 1.

**Table 1:** Clinicopathological characteristics of patients with non-small cell lung cancer (n = 100)

Variable	Long survival (n = 50)	Short survival (n = 50)
Survival (days, mean ± standard error)	1972.0 ± 78.0	351.8 ± 32.5
Age (years, mean ± standard error)	58.0 ± 1.4	60.5 ± 1.3
Gender (male: female)	40:10	41:9
Tumor stage: number (%)		
I	24 (48)	11 (22)
II	9 (18)	11 (22)
III	15 (30)	20 (40)
IV	2 (4)	8 (16)
Histology: number (%)		
Adenocarcinoma	12 (24)	24 (48)
Squamous	34 (68)	17 (34)
Alveolar cell	3 (6)	7 (14)
Large cell	1 (2)	2 (4)
Tumor grade: number (%)		
Well differentiation	2 (4)	0 (0)
Moderate differentiation	27 (54)	21 (42)
Poor differentiation	15 (30)	15 (30)
Not recorded	6 (12)	14 (28)
Lymph node metastasis: number (%)		
No	37 (74)	23 (46)
Yes	13 (26)	27 (54)

### Immunohistochemistry

Four-μm thick tissue sections were de-waxed in xylene and rehydrated through graded alcohols. Antigen retrieval was carried out using microwave at middle-to-high temperature for 8 min, low-to-high temperature for 5 min, and then cooled down at room temperature for 20 min. Mouse anti-human CD68 monoclonal antibodies (clone KP1, recognizing macrophages), mouse anti-HLA-DR monoclonal antibodies (clone LN3, recognizing M1 macrophages), and mouse anti-human CD163 antigen monoclonal antibodies (clone 10D6, recognizing M2 macrophages) were produced by Invitrogen, Carlsbad, CA, and were obtained from Zhongshan Goldenbridge Biotechnology Co., LTD., Beijing, China. Mouse IgG1 (clone NCG01, Abcam, Cambridge, MA, USA) was used as an isotype negative control.

Immunohistochemical staining of individual marker or double-staining of CD68 and CD163 or CD68 and HLA-DR was performed using the DouSP™ double-stain kit (Maxim-Bio, Fuzhou City, Fujian Province, China) according to the manufacturer's instructions. Development of red color was performed using streptavidin-peroxidase conjugate and 3-Amino-9-ethylcarbazole (AEC). Development of black-purple color was performed using streptavidin-alkaline-phosphatase conjugate and 5-bromo-4-chloro-3-indolyl phosphate (BCIP)/nitro blue tetrazolium (NBT). Sections were then counterstained with hematoxylin and mounted in an aqueous mounting medium. Tissue sections with macrophages previously stained positively were used as positive control, while tissue sections with primary antibodies replaced by mouse isotype IgG1 served as negative control. Five representative high-power fields (× 400 magnification) per tissue section were selected using an Olympus BX51 microscope (Olympus, Tokyo, Japan). Areas of the tumor islets, tumor stroma, or combination of the tumor islets and stroma were defined and measured using the Image-Pro Plus 6.0 software (Media Cybernetics, Silver Spring, MD, USA). The number of nucleated cells with positive staining for the phenotypic marker in each area was counted. Macrophage density was calculated as cell number per mm^2 ^of the tumor islets, stroma, or islets and stroma. Evaluation of the stained tissue sections was performed by two investigators who were blinded in regard to which group the specimens belonged to. Macrophage density of each case was an average of the results obtained by the two examiners.

### Statistical analysis

Statistical analysis was carried out using the Statistical Package for the Social Sciences (SPSS, version 13.0, SPSS Inc., Chicago, IL, USA). For categorical analysis, the median value of macrophage density was used as a cut-off point to dichotomize the continuous variables. The Mann-Whitney nonparametric test was used to compare between two groups. The Spearman's rank correlation coefficient was calculated to assess any potential relationship between macrophage density and patient's survival time. The Kaplan-Meier survival curves were used to look for correlation between macrophage density and patient's survival time. Statistical significance was analyzed using the log-rank test. A multivariate Cox proportional hazards model was used to estimate adjusted hazard ratios and 95% confidence intervals (CI) and to identify which form of macrophages was an independent prognostic factor. The validity of the proportional hazards assumption was assessed from log (-log [Survival]) curves. For the above comparisons, P < 0.05 was considered statistically significant.

## Results

### Patient characteristics

Among the 100 patients (Table 1) who had undergone surgery, 10 patients received additional chemotherapy after surgery. No patients received radiotherapy before or after surgery. All of the patients had complete follow-up information and the pathological diagnosis was verified by a pathologist prior to inclusion in this study. The overall cumulative survival rates were 68% for 1 year, 46% for 3 years, and 32% for 5 years. The group of patients with long survival time had survived 5.4 years on the average, whereas the group of patients with short survival time had lived only about one year (Table 1).

### Immunohistochemical detection of macrophages

**Figure 1:**
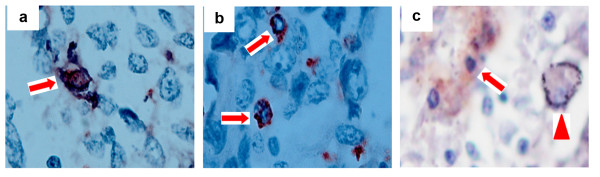
**Immunohistochemical detection of macrophages in NSCLC tumor islets**. a) The M1 macrophage double stained with the anti-CD68 (red) and anti-HLA-DR (black-purple) antibodies (arrow). b) The M2 macrophages double stained with the anti-CD68 (red) and anti-CD163 (black-purple) antibodies (arrow). c) The M1 macrophage marker HLA-DR stained black-purple (arrowhead) and the M2 macrophage marker CD163 stained red (arrow). Original magnification, × 1000 for a & b, and × 400 for c.

In order to assess whether the markers chosen actually detect different cellular subsets of macrophages, tissue sections from five patients were initially stained for CD68, CD163, or HLA-DR, alone or in combination. It was found that CD68 staining identified cells with morphological features of macrophages. Among the CD68+ macrophages, some of them stained positively for HLA-DR, a marker of the M1 macrophages (Figure 1a), whereas others stained positively for CD163, a marker of the M2 macrophages (Figure 1b). In double-staining for CD163 and HLA-DR, a majority of macrophages stained positively for either CD163 or HLA-DR (Figure 1c). Only a small percentage of macrophages (median 3.1%, range 1.2% - 8.1%) stained positively for both CD163 and HLA-DR.

### M1 and M2 macrophage densities in the tumor islets and stroma

When the M1 and M2 macrophages on the tumor sections (including the tumor islets and stroma) were counted under high-power fields, approximately 70% of macrophages were CD68+/CD163+ M2 macrophages and the remaining 30% of them were CD68+/HLA-DR+ M1 macrophages. The M1 macrophage density (approximately 70/mm^2^) in the tumor islets and stroma of patients with long survival time was significantly higher (about 4 times) than the M1 macrophage density (approximately 17/mm^2^) in the tumor islets and stroma of patients with short survival time (P < 0.001) (Table 2). The M2 macrophage density in the tumor islets and stroma of the long survival group (approximately 98/mm^2^) was not significantly different from the M2 macrophage density in the tumor islets and stroma of the short survival group (approximately 110/mm^2^) (P > 0.05). The M1/M2 ratio in the tumor islets and stroma was 0.7 in patients with long survival time and 0.2 in patients with short survival time (Table 2).

**Table 2:** Density and microlocalization of macrophages in non-small cell lung cancer

	Long survival			Short survival			
		
MΦ Form	Islets	Stroma	I + S	Islets	Stroma	I + S	[I +S] long/[I + S] short
M1	70.1 (0 - 255.3)	33.6 (0 - 257.1)	70.4 (0 - 255.7)	7.3 (0 - 74.9)	13.1 (0 - 129.9)	17.2 (0 - 132.2)	4.1
M2	77.6 (0 - 356.9)	78.4 (0 - 327.9)	97.9 (0 - 299.2)	113.4 (0 - 311.5)	79.5 (0 - 234.3)	109.5 (0 - 257.5)	0.9
M1/M2	0.9	0.4	0.7	0.1	0.2	0.2	4.5

Macrophage (MΦ) density is presented as median (range) of cell number per mm^2^. I +S represents median (range) of the macrophage density in the tumor islets and stroma, which is obtained based on macrophage number per mm^2 ^of the tumor sections and not a simple sum of the macrophage densities in the islets plus that in the stroma. [I +S] long/[I +S] short represents a ratio of the median macrophage density in the tumor islets and stroma of the long survival group versus that of the short survival group. Analyzed with Mann-Whitney nonparametric test, the M1 macrophage densities in the tumor islets, stroma, and I+S of patients with long survival time are significantly higher than those of patients with short survival time (P < 0.001, P < 0.05, and P < 0.001, respectively). The M2 macrophage densities in the tumor islets, stroma, and I+S of patients with long survival time are not significantly different from those of patients with short survival time (P = 0.526, P = 0.929, and P = 0.329, respectively). The M2 macrophage density is not significantly different from the M1 macrophage density in the tumor islets of patients with long survival time (P > 0.05). The M2 macrophage density in the tumor stroma of the long survival group and the M2 macrophage densities in the tumor islets or stroma of the short survival group are significantly higher than the corresponding M1 macrophage densities (P < 0.01).

When the M1 and M2 macrophages were assessed in the tumor islets or stroma individually, the M1 macrophage densities in the tumor islets (approximately 70/mm^2^) and stroma (approximately 34/mm^2^) of the long survival group were significantly higher than the M1 macrophage densities in the tumor islets (approximately 7/mm^2^) and stroma (13/mm^2^) of the short survival group (P < 0.001 and P < 0.05, respectively) (Table 2). The M2 macrophage densities in the tumor islets (approximately 78/mm^2^) and stroma (approximately 78/mm^2^) of the long survival group were not significantly different from the M2 macrophage densities in the tumor islets (approximately 113/mm^2^) and stroma (80/mm^2^) of the short survival group (P > 0.05) (Table 2). The M1/M2 ratios in the tumor islets (a ratio of 0.9) and stroma (a ratio of 0.4) of patients with long survival time were about 9 and 2 times of the corresponding M1/M2 ratios in the tumor islets (a ratio of 0.1) and stroma (a ratio of 0.2) of patients with short survival time, respectively (Table 2).

### Correlation between the macrophage density and survival time

**Figure 2:**
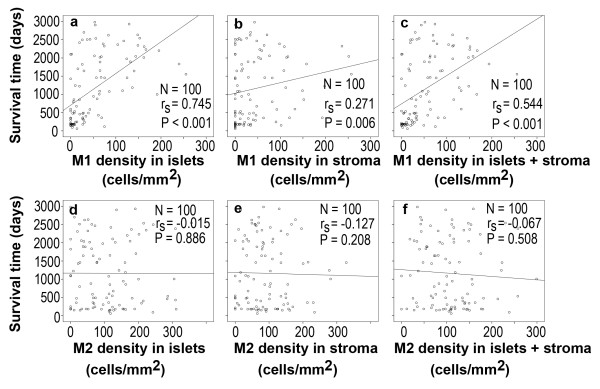
**Scatter plots of the macrophage density versus patient's survival time**. r_s _represents the Spearman's rank correlation coefficient.

Scatter plots of the macrophage density versus survival time were shown in Figure 2. The Spearman's rank correlation coefficient (r_s_) was calculated to assess any potential relationship between the macrophage density and patient's survival time. We found that the M1 macrophage densities in the tumor islets, stroma, or islets and stroma were positively associated with patient's survival time, with r_s _= 0.745 (P < 0.001), 0.271 (P < 0.01), and 0.544 (P < 0.001), respectively. In contrast, there was no association between the M2 macrophage densities and patient's survival time (P > 0.05).

**Figure 3:**
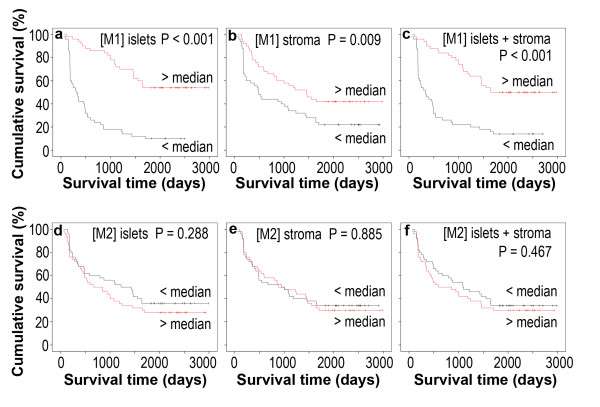
**Kaplan-Meier survival curves**. One hundred patients (N = 100) are divided into two groups with the macrophage densities above or below the median value. P values are obtained in comparisons of the two groups through a univariate analysis using the log-rank test. [M1] and [M2] represent the M1 macrophage density and M2 macrophage density, respectively.

In order to assess whether there is any value of the macrophage density in predicting prognosis, the median value of the macrophage density was used as a cut-off point to dichotomize the 100 patients into a group with a macrophage density above the median and a group with a macrophage density below the median. We found that patients with above-the-median M1 macrophage density in the tumor islets had a 1-year survival rate of 94%, 3-year survival rate of 74%, and 5-year survival rate of 54%, which were significantly higher than the corresponding survival rates (42%, 18%, and 10%, respectively, P < 0.001) in patients with below-the-median M1 macrophage density in the tumor islets (Table 3). Patients with above-the-median M1 macrophage density in the tumor stroma had a 1-year survival rate of 78%, 3-year survival rate of 58%, and 5-year survival rate of 42%, which were significantly higher than the corresponding survival rates (58%, 34%, and 22%, respectively, P < 0.01) in patients with below-the-median M1 macrophage density in the tumor stroma. Patients with above-the-median M1 macrophage density in the tumor islets and stroma had a 1-year survival rate of 90%, 3-year survival rate of 70%, and 5-year survival rate of 50%, which were significantly higher than the corresponding survival rates (46%, 22%, and 14%, respectively, P < 0.001) in patients with below-the-median M1 macrophage density in the tumor islets and stroma (Table 3). In contrast, the M2 macrophage densities in the tumor islets, stroma, or islets and stroma had no statistically significant association with patient's survival time. The Kaplan-Meier survival curves further illustrated association of the M1 but not M2 macrophage densities with patient's survival time (Figure 3).

**Table 3:** Correlation between the density and microlocalization of macrophages and survival time in non-small cell lung cancer patients

		Survival rate (%)			P (univariate)	Cox regression	
				
Density and microlocalization	n	1-yr	3-yr	5-yr		Hazard ratio (95% CI)	P (multivariate)
M1 density in islets						0.982 (0.975 - 0.989)	< 0.001
Above median	50	94	74	54	< 0.001		
Below median	50	42	18	10			
M1 density in stroma						1.001 (0.992 - 1.009)	0.889
Above median	50	78	58	42	0.009		
Below median	50	58	34	22			
M1 density in islets + stroma						1.001 (0.986 - 1.015)	0.943
Above median	50	90	70	50	< 0.001		
Below median	50	46	22	14			
M2 density in islets						0.997 (0.989 - 1.006)	0.535
Above median	50	68	38	28	0.288		
Below median	50	68	54	36			
M2 density in stroma						0.997 (0.991 - 1.004)	0.430
Above median	50	68	48	30	0.885		
Below median	50	68	44	34			
M2 density in islets + stroma						1.007 (0.994 - 1.021)	0.290
Above median	50	64	42	30	0.467		
Below median	50	72	48	34			

In order to determine whether the macrophage density is independently associated with patient's survival time, the multivariate Cox proportional hazards analysis was used. Tumor stage, histology and grade were included in the multivariate analysis along with the macrophage density, because we found that there was no statistically significant association between the M1 macrophage density and these clinicopathological characteristics (P > 0.05). Status of lymph node metastasis was excluded because the patients with lymph node metastasis had statistically lower M1 macrophage density in the tumor islets than the patients without lymph node metastasis (P < 0.05). We found that the M1 macrophage density in the tumor islets was a positive independent predictor of patient's survival time (hazard ratio 0.982, 95% CI 0.975 - 0.989, P < 0.001). The M1 macrophage densities in the tumor stroma or tumor islets and stroma, or any M2 macrophage densities had no statistically significant association with patient's survival time in the multivariate analysis (Table 3).

## Discussion

Macrophage is a major component of inflammatory infiltrate of tumors [11,12]. Tumor-associated macrophages (TAMs) have complex dual functions in terms of their anti-tumor or pro-tumor effects. This functional complexity is related to the heterogeneity of macrophage population that hold a continuum of diverse functional states. At one end of the continuum are the classically activated M1 macrophages that produce effector molecules such as reactive oxygen intermediates, reactive nitrogen intermediates, and TNFα, to limit tumor growth. At the other end are the alternatively activated M2 macrophages that promote tumor growth and metastasis by secretion of matrix-degrading enzymes, angiogenic factors and immunosuppressive cytokines/chemokines [5]. The balance of these macrophage forms determines the anti- or pro-tumor effects of the macrophage population [11].

Heterogeneity of macrophages may account for part of the controversies in regard to TAMs' role in prognosis. For example, Chen et al reported that TAMs are negatively associated with survival in NSCLC patients [13]. Toomey et al found that there is no association between macrophage number and prognosis of NSCLC [14]. On the other hand, Welsh et al found that the macrophage density in the tumor islets is positively associated with patient's survival [4]. Our previous study showed that the macrophage density in the tumor islets is positively associated with survival, whereas the macrophage density in the tumor stroma is negatively associated with survival [10]. We hypothesized that assessment of the M1 versus M2 macrophage density in the tumor islets and stroma may provide new insights into understanding the role of TAMs in prognosis of NSCLC.

In this study, we found that in 100 cases of non-small cell lung cancer, the M2 macrophage is the predominant form of TAMs, consisting of 70% of the overall macrophage population. In comparison between the long survival and short survival groups of patients, there is no significant difference with respect to the M2 macrophage densities in the tumor islets, stroma, or islets and stroma. In contrast, the M1 macrophage densities in the tumor islets, stroma, or islets and stroma are significantly higher in the long survival group than in the short survival group. These findings imply that the M1 macrophage density may be associated with patient's survival. Indeed, using the Spearman's rank correlation coefficient analysis, we confirmed that the M1 macrophage densities in the tumor islets, stroma, or islets and stroma are positively associated with patient's survival time. Furthermore, using the median value of the M1 macrophage density as a cut-off point, patients with above-the-median M1 macrophage density in the tumor islets, stroma, or islets and stroma had significantly higher cumulative survival rates, compared to patients with below-the-median M1 macrophage density in a univariate analysis. However, in a multivariate analysis, only the M1 macrophage density in the tumor islets remains as an independent predictor of patient's survival time. These results suggest that assessment of the M1 macrophage density in the tumor islets can be very helpful in predicting survival time of patients with NSCLC.

Our results are consistent with a recent report that the M1 macrophage density in the tumor islets is positively associated with extended survival of NSCLC patients [9]. What differs between the two studies is that we do not observe any statistically significant difference in terms of the M2 macrophage densities between the long survival and short survival groups, whereas the other study [9] found an increase of the M2 macrophage density in the tumor islets of the extended survival group. Both studies used the same clone (10D6) of mouse anti-human CD163 antigen monoclonal antibodies and similar techniques to detect the M2 macrophage. We speculate that difference in patient populations and evaluation protocols may be the main reasons for the discrepancy. Nevertheless, these studies suggest that immune responses, particularly infiltration of the M1 macrophages into the tumor islets, may play a crucial role in preventing progression of non-small cell lung cancer. The putative biological mechanisms may include direct effects of reactive oxygen/nitrogen intermediates and TNFα released by the M1 macrophages in close proximity to the tumor cells, and/or indirect effects through activation and recruitment of cytotoxic T cells [15]. It is speculated that any pharmacological interventions to induce differentiation of the M1 macrophages and/or to promote infiltration of the M1 macrophages into the tumor islets would provide therapeutic benefits to patients with non-small cell lung cancer. Future studies are needed to confirm this speculation.

The limitation of this study is that the markers used are not very specific. Although CD68 has been widely used to identify macrophages [9,16-18]], it has been found in immature CD1a-positive dendritic cells [19,20]. HLA-DR has been found to be expressed in the peripheral lymphocytes [21] and CD163 is also expressed in some dendritic cells [22]. Therefore, there is a possibility that some cells as identified by these three markers are not macrophages. Ideally, the cells should be stained with multiple markers. However, multiple staining would be technically challenging in handling the archived paraffin-embedded tissues.

## Conclusions

This study demonstrates that the tumor-associated macrophages in non-small cell lung cancer contain two distinct forms, a CD68+/HLA-DR+ M1 form and a CD68+/CD163+ M2 form. The M1 and M2 macrophages consist of 30% and 70% of TAMs in NSCLC, respectively. The M1 macrophage densities in the tumor islets, stroma, or islets and stroma are significantly higher in patients with an average of 5-year survival, compared to patients with an average of 1-year survival. The M1 macrophage density in the tumor islets is an independent factor that can predict patient's survival time. The M2 macrophage density is not associated with patient's survival time.

## Competing interests

The authors declare that they have no competing interests.

## Authors' contributions

JM performed immunohistochemistry, evaluated the stained slides, performed statistical analysis, and drafted the manuscript. LL and GC designed and supervised the collection of data. NY collected the clinicopathological data. FD obtained the slides and evaluated the stained slides. ZY analyzed and interpreted the data and prepared the tables, figures, and manuscript text. All authors participated in manuscript preparation and approved the final version prior to submission.

## Pre-publication history

The pre-publication history for this paper can be accessed here:

http://www.biomedcentral.com/1471-2407/10/112/prepub
